# Crystal Structure and Biochemical Analysis of a Cytochrome P450 Steroid Hydroxylase (*Ba*CYP106A6) from *Bacillus* Species

**DOI:** 10.4014/jmb.2211.11031

**Published:** 2022-12-12

**Authors:** Ki-Hwa Kim, Hackwon Do, Chang Woo Lee, Pradeep Subedi, Mieyoung Choi, Yewon Nam, Jun Hyuck Lee, Tae-Jin Oh

**Affiliations:** 1 Department of Life Science and Biochemical Engineering, Sunmoon University, Asan 31460, Republic of Korea; 2 Research Unit of Cryogenic Novel Materials, Korea Polar Research Institute, Incheon 21990, Republic of Korea; 3 Department of Polar Sciences, University of Science and Technology, Incheon 21990, Republic of Korea; 4 Department of Pharmaceutical Engineering and Biotechnology, Sunmoon University, Asan 31460, Republic of Korea; 5 Genome-based BioIT Convergence Institute, Asan 31460, Republic of Korea

**Keywords:** Crystal structure, cytochrome P450, steroid hydroxylase, X-ray crystallography

## Abstract

Cytochrome P450 (CYP) is a heme-containing enzyme that catalyzes hydroxylation reactions with various substrate molecules. Steroid hydroxylases are particularly useful for effectively introducing hydroxyl groups into a wide range of steroids in the pharmaceutical industry. This study reports a newly identified CYP steroid hydroxylase (*Ba*CYP106A6) from the bacterium *Bacillus* sp. and characterizes it using an in vitro enzyme assay and structural investigation. Bioconversion assays indicated that *Ba*CYP106A1 catalyzes the hydroxylation of progesterone and androstenedione, whereas no or low conversion was observed with 11β-hydroxysteroids such as cortisol, corticosterone, dexamethasone, and prednisolone. In addition, the crystal structure of *Ba*CYP106A6 was determined at a resolution of 2.8 Å to investigate the configuration of the substrate-binding site and understand substrate preference. This structural characterization and comparison with other bacterial steroid hydroxylase CYPs allowed us to identify a unique Arg295 residue that may serve as the key residue for substrate specificity and regioselectivity in *Ba*CYP106A6. This observation provides valuable background for further protein engineering to design commercially useful CYP steroid hydroxylases with different substrate specificities.

## Introduction

Steroids are biological compounds that play key roles in the body, including controlling metabolism and the immune system, synthesizing muscle and bone, and maintaining homeostasis [1-3]. Steroid-based drugs are one of the most widely used clinical drugs to treat inflammation, rheumatoid arthritis, cancer, and allergic reactions and are also used as convulsants and contraceptives [4-7]. Since hydroxylated steroids generally exhibit higher biological activity than less polar steroids, research on hydroxylated steroid production methods has focused on and expanded from a chemical method that has disadvantages in terms of time and cost to an eco-friendly biocatalytic method using a bacteria-derived enzyme such as Cytochrome P450 (CYP) [5, 8].

CYP is a large family of heme-containing monooxygenases that catalyzes various reactions in secondary metabolite and natural product biosynthesis and xenobiotic metabolism in humans [9]. Plants have hundreds of CYPs per species, which are used to synthesize substrates, such as alkaloids, terpenes, and flavonoids [10]. Among them, microbial CYPs catalyze a broad spectrum of substrates and are more popular as biocatalysts industrially because of their ease of heterologous expression compared with mammalian and plant CYP [11-13]. Accordingly, the bioconversion of steroids using microbial CYPs has been addressed. For example, P450_lun_ from *Curvularia lunata* was applied to produce cortisol from 11-deoxycortisol [14, 15]. CYP105A2 from *Pseudonocardia autotrophica* and CYP105A1 from *Streptomyces griseolus* were also used for transforming vitamin D_3_ to 1α,25-dihydroxy-vitamin D_3_, which can be used to treat vitamin D_3_ deficiency and metabolic disorder such as hypocalcemia, psoriasis, and osteoporosis [16-18].

The CYP106 and CYP109 families hydroxylate various types of steroids and terpenoids. For example, CYP106A1 from *Bacillus megaterium* DSM319 showed hydroxylation of a triterpene, 11-keto-β-boswellic acid (KBA), at the 7β- and 15α-positions using the whole cell system [19]. The well-studied *Bm*CYP106A2 from *B. megaterium* ATCC 13368 also hydroxylated the 15β position of 3-oxo-Δ^4^-steroids such as progesterone, corticosterone, testosterone, and androstenedione, and 7β position of dehydroepiandrosterone (DHEA) and pregnenolone [20-22]. Additionally, CYP109A2, another CYP from *B. megaterium* DSM319, hydroxylated vitamin D_3_ resulting in 25-hydroxyvitamin D_3_ [23]. Recently, our group showed that *Ba*CYP106A2 from *Bacillus* sp. PAMC 23377 converts 4-androstenedione or nandrolone into 15β-hydroxy-androstenedione or 7β,15β-dihydroxyandrostenedione, respectively [24].

Although these members are similar, with approximately 30% or greater sequence identity, the substrate preferences and hydration site regions of each CYP differ. This indicates that sequential alignment does not distinguish the characteristics of CYPs, and biochemical and structural investigations of each CYP are necessary to understand the detailed mechanisms of bacterial CYPs for steroid hydroxylation.

In the present study, we report the biochemical characterization and crystal structure of *Ba*CYP106A6—at a 2.8-Å resolution—from multi-*Bacillus* species isolated from soil. Accordingly, the results provide valuable insights for further protein engineering to design commercially applicable CYP steroid hydroxylases with different substrate specificities.

## Materials and Methods

### Materials

The substrates were purchased from Sigma-Aldrich (Korea) and Tokyo Chemical Industry Co. Ltd. (Japan). All the enzymes, including Taq polymerase and restriction enzymes, were obtained from Takara Clontech (Korea). All other chemicals and solvents used were of the highest commercially available grade (ACS, HPLC grade; Fisher Scientific, Korea). Ampicillin, α-aminolevulinic acid, NADPH, catalase, glucose-6-phosphate dehydrogenase, and glucose-6-phosphate, the redox partners of spinach FDX and FDR, were purchased from Sigma-Aldrich. Isopropyl 1-thio-β-d-galactopyranoside (IPTG) and kanamycin were purchased from Duchefa Biochemie (Korea).

### Over-expression and Purification of *Ba*CYP106A6

*Ba*CYP106A6-pET28a was introduced into *Escherichia coli* C41(DE3) and inoculated into Luria–Bertani (LB) medium supplemented with ampicillin (100 μg/ml). Thereafter, the seed culture was transferred to LB containing 0.5 mM FeCl_3_·6H_2_O, 1 mM 5-aminolevulinic acid hydrochloride (5-ALA), 100 μg/ml ampicillin at 37°C. The culture was induced with 0.5 mM IPTG when the optical density at 600 nm (OD_600_) reached 0.6–0.8. After 72 h of incubation at 20°C, the cells were harvested and suspended in 50 mM potassium buffer (pH 7.4). The cell extract was sonicated and centrifuged at 10,000 rpm for 20 min to obtain the soluble protein solution. Next, the protein solution was mixed with Co^2+^ resin solution and eluted with elution buffer (potassium phosphate buffer, pH 7.4) containing 20, 100, and 250 mM imidazole. The eluted fraction containing the protein was concentrated using a 30 K Amicon Ultra centrifugal filter (Millipore, Ireland).

### Enzyme Analysis and Substrate-Binding Assay

The concentration of the purified *Ba*CYP106A6 was calculated using a UV-visible spectrophotometer (Biochrome Libra, UK). The concentration of *Ba*CYP106A6 was determined by CO-bound reduced difference spectroscopy using an extinction coefficient of 91 mM^-1^cm^-1^. CO difference spectroscopy measurements were performed according to the method of Omura and Sato [25]. Protein purity and molecular weight were analyzed using SDS-PAGE.

To determine the degree of steroid substrate-binding to a specific enzyme, spin-shift states were identified using a UV-visible spectrophotometer and tandem quartz cuvettes. The sample cuvette chambers were filled with a total volume of 1 ml in 50 mM potassium buffer (pH 7.4), including purified protein diluted to 1 μM and substrate by concentration, and the standards were prepared without substrate. The substrates were dissolved in DMSO at a storage concentration of 40 mM, diluted in the range of 0–500 μm and used for the assay. The UV-visible absorbance spectrum was measured between 350 and 500 nm until no spectral changes were observed. The equilibrium *K_d_* of *Ba*CYP106A6 was calculated using a quadratic equation by titrating the substrate concentration until saturation:

$$\Delta A=\Delta A\max\times \frac{[E]+[S]+Kd-\sqrt{{\left([E]+[S]+Kd\right)}^{2}-4[E][S]}}{2[E]}$$

where [E] and [S] are the concentrations of the enzyme and substrate, respectively, *K_d_* is the binding constant, ΔAmax is the maximal absorption shift, and ΔA is the peak-to-trough ratio [26]. The dissociation constant (*K_d_*) of *Ba*CYP106A6 was determined by titrating different concentrations of the substrates until saturation.

### In vitro Biotransformation Assays of *Ba*CYP106A6 Activity

For the in vitro assay, two steroid substrates were used: progesterone and androstenedione. A stock solution (100 mM) of the substrate was dissolved in DMSO and stored until use. The in vitro reaction was carried out in a total volume of 250 μl in 50 mM potassium buffer (pH 7.4) consisting of 10 μM *Ba*CYP106A6, 25 μg FDX, 0.1 U FDR, 100 μM steroid substrates, 5 mM MgCl_2_, 100 μg/ml catalase, 1 mM NADPH, 1 U glucose-6-phosphate dehydrogenase (G6P-DH), and 10 mM glucose-6-phosphate. The reaction was initiated by adding 1 mM NADPH. Reaction mixtures were incubated at 30°C for 2 h, extracted twice with an equal volume of ethyl acetate, and then dried completely with nitrogen gas. Finally, the dried samples were dissolved in a 6:4 (acetonitrile/water) solution for HPLC analysis.

### HPLC and NMR Analysis

HPLC analyses for product separation were performed using an Agilent 1100 series system (G1311A Quaternary pump, G1379A Solvent degasser, G1315B Diode array detector, and G1313A Standard autosampler; Agilent Technologies, USA). This device was connected to a reversed-phase C_18_ GP column (4.6 × 250 mm, 5 μm; Mightysil; Kanto Chemical, Japan), and the analysis temperature was maintained at 40°C. The mobile phase was mixed with two solvents, water (A) and acetonitrile (B), at a rate of 1 ml·min^−1^. The HPLC system started with acetonitrile and water at a ratio of 15:85, increased to 50:50 for 8 min, and then to 90:10 for 18 min. The ratio was maintained for 19 min, reduced to 15:85 for 21 min, and finally, ran for 25 min. To detect the substrate and product, the UV detector was set to 242 or 245 nm. Mass analysis was performed using quadrupole time-of-flight/electrospray ionization mass spectrometry in the positive ion (+) mode using ultra-performance liquid chromatography (SYNAPT G2‐S/ACUITY; Waters Corp., USA). The products isolated from the steroids were analyzed using a 700 MHz NMR spectrometer (Korea Basic Science Institute, Korea). For ^1^H, ^13^C NMR, HMBC, HSQC, COSY, and ROESY, 7.3 and 15 mg progesterone and androstenedione products, respectively, were dissolved in 1 ml CDCl_3_.

### Crystallization and Data Collection

Initial crystallization screening was conducted using a TTP Labtech Mosquito LCP Crystallisation Robot (TTP Labtech, UK) with commercially available screening kits, such as MCSG1-4 (Molecular dimensions, UK), Index, and SaltRx (Hampton Research, USA). The sitting drop vapor-diffusion method was performed at 293 K in 96-well plates (Emerald Bio, , USA). A 200-nL protein solution and an equal volume of reservoir solution were mixed and equilibrated against 80 μl of reservoir solution. *Ba*CYP106A6 crystals were grown within 2 days under 0.2 M ammonium citrate dibasic and 25% (w/v) PEG 3350 condition. To obtain larger crystals, crystallization conditions were optimized using the hanging drop vapor-diffusion method at 293 K in 24-well plates. Each drop, consisting of 1 μl of protein solution and 1 μl of reservoir solution, was equilibrated against 500 μl of reservoir solution. Optimized crystals appeared after 2 days under 0.18 M ammonium citrate dibasic and 22% (w/v) PEG 3350 condition at 293 K. Because of fragileness, a single *Ba*CYP106A6 crystal was directly mounted without soaking in a cryoprotection agent. The 2.8 Å resolution of the diffraction dataset was obtained on a BL-5C beamline at the Pohang Accelerator Laboratory (Korea). The dataset containing 360 images with an oscillation range of 1° rotation was indexed, integrated, and scaled using the program *HKL*-2000 [27].

### Structure Determination and Refinement

The crystal structure of *Ba*CYP106A6 was determined by the molecular replacement method using the MOLREP program from the CCP4i suite. The crystal structure of CYP106A2 from *B. megaterium* (PDB code 5IKI; sequence identity, 65%) was used as the search model [28]. The Matthews coefficient calculation result predicted that two molecules are contained in the asymmetric unit, with a Matthews coefficient of 2.50 Å^3^Da^-1^ and solvent content of 50.79 %. Next, the initial model was iteratively rebuilt using Coot [29], refined REFMAC5 [30], and phenix.refine [31]. The final model had an *R*_cryst_ value of 0.22 and *R*_free_ value of 0.27. Model quality was checked using MolProbity [32]. Detailed refinement statistics are presented in Table 1. All graphical structural representations were generated using PyMOL [33]. The coordinates and structural factors of *Ba*CYP106A6 were deposited in the Protein Data Bank RCSB under accession code 8HG9.

### Cloning and Construction of Recombinant Plasmids

The gene for *Ba*CYP106A6 was amplified by PCR using the genomic DNA of *Bacillus* sp. as the template. The PCR primers used were 5′- GGA TCC ATG TTG AAA GAA GTC ATT CC-3′ (BamHI site underlined) as the forward and 5′-CTC GAG TCC TTA CTT ATA CAC GTT CA-3′ (XhoI site underline) as the reverse primers. The fragment was digested with restriction enzymes and ligated into a pET28a (+) vector to construct *Ba*CYP106A6-pET28a. Sequence analyses for cloning were performed by Macrogen Inc. (Korea).

## Results and Discussion

### Purifcation and Characterization of *Ba*CYP106A6

To characterize *Ba*CYP106A6, His-tagged *Ba*CYP106A6 was successfully expressed and purified in a soluble form in *E. coli* host cells. Two-step purification yielded *Ba*CYP106A6 with high purity (> 95%). SDS-PAGE analysis showed a single homogeneous band of purified proteins at the expected molecular weight of 47.1 kDa (Fig. S1A). Purified *Ba*CYP106A6 induces a type I spin-shift in the resting state. UV-visible absorption spectroscopy of *Ba*CYP106A6 revealed a significant heme Soret peak at 417 nm in the substrate-free oxidized form. This peak could be shifted to 448 nm in the reduced CO-bound form, which is a characteristic feature of the cysteine-thiolate-ligated heme of CYP in a Fe (II)-CO complex (Fig. S1B) [34-36].

### Substrate-Binding and Steroid Assays Using *Ba*CYP106A6

**Fig. 1:**
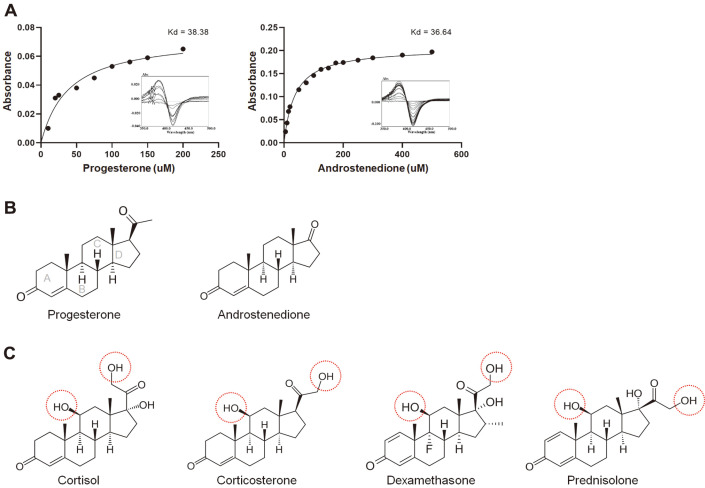
Steroid affinity test on *Ba*CYP106A6. (**A**) Type I shift spectra and *K*_d_ values using progesterone and 4- androstenedione. A total of six substrates were tested for *Ba*CYP106A6 binding. Progesterone and androstenedione showed binding affinity, whereas corticosterone, cortisol, dexamethasone, and prednisolone did not show consistent data for affinity calculation. (**B**) Steroids with a *Ba*CYP106A6-associated conversion rate > 20% were considered active steroids. (**C**) Steroids with a *Ba*CYP106A6-associated conversion rate < 20% were considered less active steroids. The conversion rate of steroids was analyzed by HPLC after 15 min incubation at 20°C.

The binding of steroids to P450 causes a type I spectral shift due to the substitution of axial water molecules in the heme iron coordination sphere [20], revealing maximum and minimum spectral values of ~390 and ~420 nm, respectively. The dissociation constant (*K_d_*) of CYP was determined by titrating different concentrations of substrates until saturation. Based on this characteristic, we conducted substrate-binding experiments with different steroidal substrates using differential spectroscopy before conducting in vitro conversions. As shown in Fig. 1A, the binding assay demonstrates that two of the six steroidal substrates could bind *Ba*CYP106A6. The equilibria *K_d_* of *Ba*CYP106A6 for progesterone and androstenedione were 38.38 and 36.64 μM, respectively (Figs. 1A and 1B). In contrast, cortisol, corticosterone, dexamethasone, and prednisolone showed no active or minor type I spin states, even at high substrate concentrations (Fig. S2). Therefore, the tested steroids could be divided into two groups based on their affinity: active and less active steroids. Interestingly, the less active steroid group had a hydroxyl group at the 11β position, while the active steroid group interacting with *Ba*CYP106A6 did not have a hydroxyl group at the corresponding site (Figs. 1B and 1C). Moreover, the less active steroid group had an additional methanol group at the acetyl group linked at the 17β position. Therefore, we hypothesized that the hydroxyl group and the side chain length at the 17β position are critical factors determining substrate affinity to *Ba*CYP106A6.

**Fig. 2:**
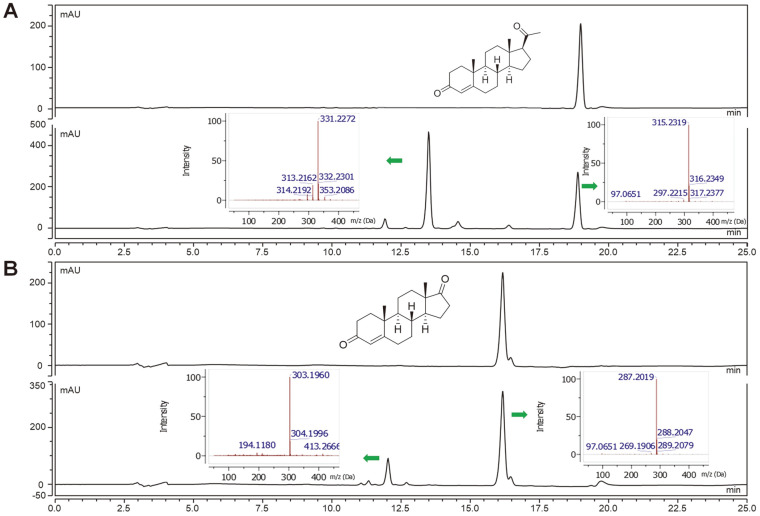
In vitro HPLC and LC/MS analysis of *Ba*CYP106A6. The HPLC spectra of progesterone (**A**) and androstenedione (**B**) with their corresponding *Ba*CYP106A6 catalyzed reaction mixture. Each substrate generated a monohydroxylated product. The masses of the substrate and monohydroxylated product are shown by the arrows.

**Fig. 3:**
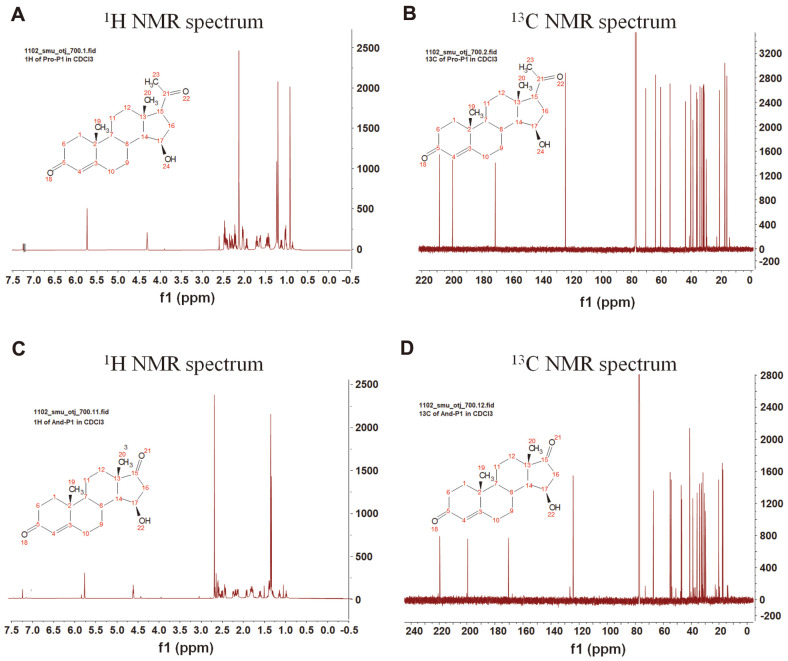
Nuclear magnetic resonance (NMR) analysis. ^1^H and ^13^C NMR analyses of hydroxylated products of progesterone (**A, B**) and androstenedione (**C, D**).

Next, we performed a bioconversion reaction of progesterone and androstenedione using recombinant *Ba*CYP106A6. Before the analysis, we confirmed that FDX/FDR could be a redox partner of *Ba*CYP106A6. As expected from the binding assay, the results indicated that *Ba*CYP106A6 could catalyze the hydroxylation of these steroids. In the case of progesterone, after incubation with *Ba*CYP106A6, the retention time at λmax:245 nm was shifted to ~13.41 min, which is equivalent to the mass for the molecular formula C_21_H_30_O_4_ for [M + H]+ m/z+ ~331.2273, confirming the monohydroxylated progesterone product (Fig. 2A). Similarly. The analysis of androstenedione from the reaction mixture showed monohydroxylated products as observed by mass [M + H]+ m/z+ ~303.1960 (Fig. 2B). Calculating the retention area showed that progesterone showed the highest conversion (~70%), while androstenedione showed a 30% conversion from the substrate after 15 min of incubation. Furthermore, NMR analysis of the chemical structures of the hydroxylated products of steroids indicated that the C15 region was the hydroxylation site by *Ba*CYP106A6 with high regio- and stereoselectivity (Fig. 3). By comparing the NMR spectroscopic data reported in the literature, the structures of the products were determined to be 15β-hydroxy progesterone and 15β-hydroxy androstenedione [37, 38].

### Structure Determination and Overall Structure of *Ba*CYP106A6

The crystal structure of *Ba*CYP106A6 was determined at a 2.8-Å resolution using X-ray crystallography. The phase problem was solved using the crystal structure of CYP106A2 (PDB code:5IKI) via the molecular replacement method. Next, the coordinates were refined as the values for *R*_work_ and *R*_free_ of the final model were 22 and 27%, respectively (Table 1). Due to an ambiguous electron density map, all amino acids were built except for the four N-terminal residues and residues from the 74–84 and 178–181 regions. The final model of *Ba*CYP106A6 contained two monomers in the asymmetric unit, with the heme cofactor located at the center of each monomer. The two monomers form 2-fold rotational symmetry with a small interface area, which seems too small to form a realistic dimeric interface, indicating that *Ba*CYP106A6 exists as a monomer. Consistent with this, size-exclusion chromatography analysis indicated that *Ba*CYP106A6 is a stable monomer in solution (Fig. S3).

**Fig. 4:**
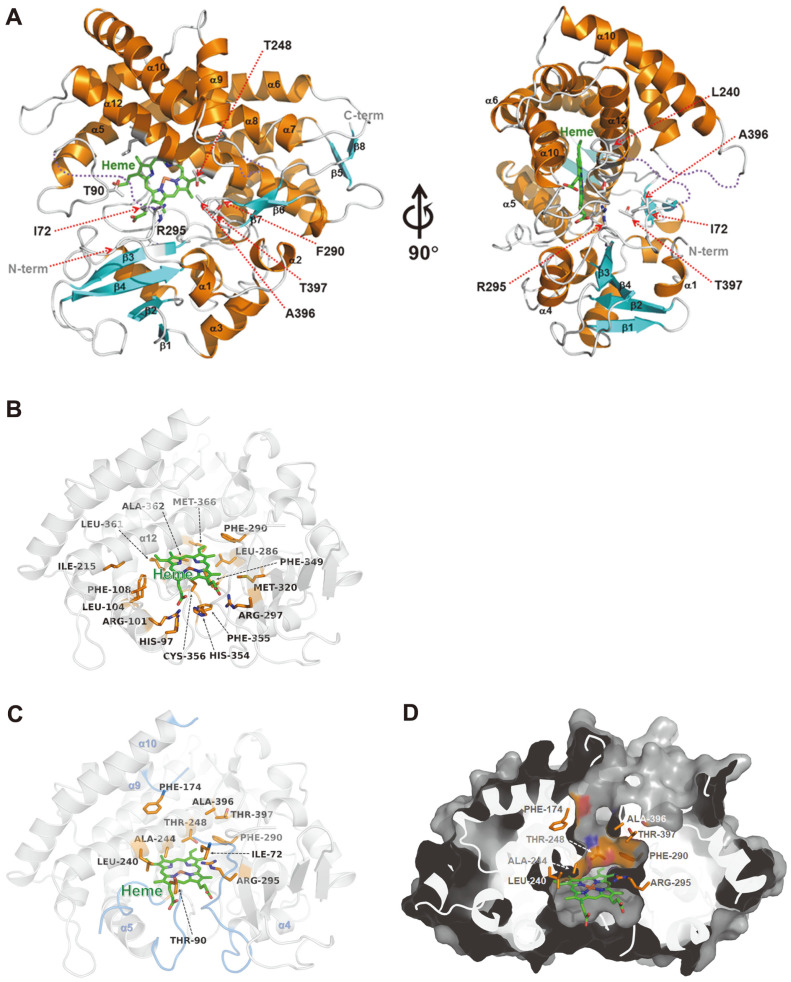
The overall structure and active site of *Ba*CYP106A6. (**A**) The overall structure of *Ba*CYP106A6 is presented as a ribbon diagram in the front view (left) and a 90° rotated view (right). The α-helices and β-strands are colored orange and cyan, respectively. The bound heme molecules are represented by a stick model and colored green. (**B**) Heme-binding motif of *Ba*CYP106A6. Hydrophobic residues surrounding the heme molecule and residues interacting with carboxyl groups of heme are presented as an orange stick model. The heme molecule is represented by a green stick model. (**C**) The putative substratebinding pocket of *Ba*CYP106A6. Several specific residues comprising substrate-binding pockets are presented as orange stick models. Disordered regions of the α4–α5 and the α9–α10 loops are colored marine. (**D**) Surface representation of *Ba*CYP106A6 shown in the same orientation as

The monomeric structure of *Ba*CYP106A6 consists of 17 α-helices and eight β strands and exhibits a typical fold seen in CYP monooxygenases with a centered heme cofactor molecule (Fig. 4A). Structural analysis of *Ba*CYP106A6 revealed a heme cofactor-binding site and putative substrate-binding residues. The heme molecule is tightly bound to the central region of the *Ba*CYP106A6 structure and is surrounded by α5, α12, α14, and α16 helices. The Cys356 residue is involved in the coordination of heme iron. The carboxyl groups of heme interact with the side chains of His97, Arg101, Arg297, and His354. The nonpolar part of the heme is surrounded by hydrophobic residues Leu104, Phe108, Ile215, Leu286, Phe290, Met320, Phe349, Phe355, Leu361, Ala362, and Met366. These conserved heme-binding features are typical of CYP monooxygenases (Fig. 4B). The putative substrate-binding pocket is located directly above the heme molecule and consists of Ile72, His80, Asn88, Thr90, Leu240, Ala244, Thr248, Arg295, Ala396, and Thr397 (Figs. 4C and 4D).

### Substrate Selectivity of BaCYP106A6

The binding mode of steroids was analyzed to better understand the substrate selectivity of *Ba*CYP106A6. First, because the structure has unconnected residues between the 74–84 and 178–181 regions, the complete structure was generated by the SWISS modeling server based on the *Ba*CYP106A6 structure as a template [39]. The steroids were superposed with abietic acid from the CYP106A2 structure (PDB:5IKI) and corticosterone from the CYP109E1 structure (PDB:5L91) in the putative binding pocket, and energy minimization was conducted using the YASARA Energy Minimization Server [40]. Energy minimization with a substrate-complexed structure avoids substrate hindrance or residual crashes between steroids and proteins. The results showed that the distances between the iron of heme and the C15 of progesterone and androstenedione were 4.3 and 3.8 Å, respectively, indicating that the orientation and distance of steroids are in a feasible conformation.

**Fig. 5:**
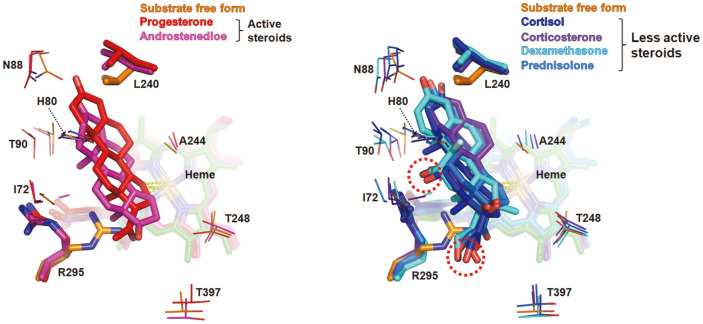
Overlay of steroids as obtained by superposition onto *Ba*CYP106A6. The active (left) and less active steroids (right) occupy the substrate-binding pocket depicted in red and blue series colors. Energy-minimized structures and heme molecules are colored the same for each steroid. The hydroxyl groups at 11β and 17β of the less active steroids, marked with red dotted circles, are placed near an Arg295.

Compared with the substrate-free structure, the direction of the side chain of Leu240 and Arg295 was mainly changed among residues interacting with substrates in steroid-complexed structures. The side chain of Leu240 leans to the heme molecule in the substrate-free structure, and the side chain is pushed away by the C4 of the steroids upon steroid binding, generating a hydrophobic interaction. In the case of Arg295, its side chain protrudes inside the binding pocket and occupies a large volume of the substrate-binding pocket in a substrate-free structure (Fig. S4). However, in the steroid-binding mode, Arg295 was tilted opposite the active site (Fig. 5). These changes imply that Leu240 and Arg295 may be critical residues that recognize specific *Ba*CYP106A6 substrates. Moreover, Arg295 was closely located to the hydroxyl group at the 11β position of the less active steroid group and was positioned in the hydrogen interaction range of 3–5 Å. Another possible interaction is between Arg295 and the 17β side chain. It is worth noting that we speculated that the hydroxyl group at 11β and/or the 17β position side chain were critical points distinguishing the types of steroids from the substrate-binding assay. Since only less active steroids have a hydroxyl group at the 11β position and this hydroxyl group is likely to interact with Arg295, we provisionally concluded that the interaction of Arg295 with negatively charged functional groups, such as the hydroxyl group at the 11β and/or 17β positions of less active steroids, may interfere with the less active steroids that are suited to the active site, resulting in low activity.

### Comparison of Substrate-Binding Pocket between CYP106 and CYP109 Proteins

**Fig. 6:**
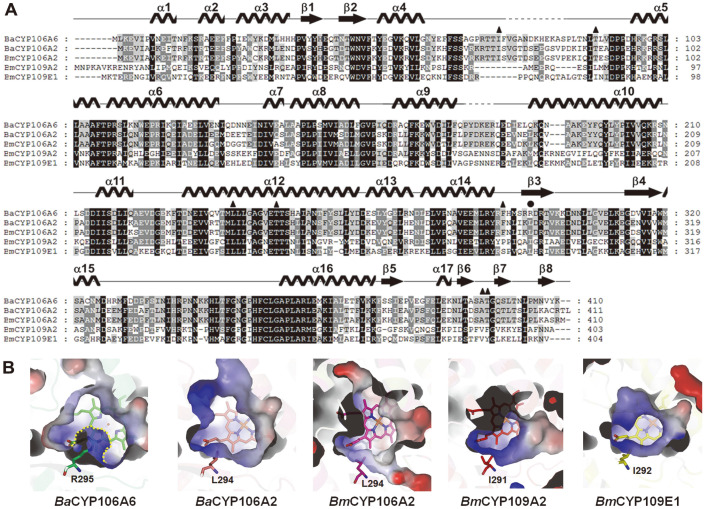
Multiple sequence alignment and binding mode of steroids. (**A**) Multiple sequence alignment of *Ba*CYP106A2 with other homolog CYPs, *Ba*CYP106A2 (*Bacillus* sp. PAMC23377; PDB code 5XNT), *Bm*CYP106A2 (*B. megaterium*; UniProtKB code: Q06069; PDB code 4YT3), *Bm*CYP109A2 (*B. megaterium*; UniProtKB code: D5DF88; PDB cod 5OFQ), and *Bm*CYP109E1 (*B. megaterium*; UniProtKB code: D5DKI8; PDB code 5L90). Secondary structures of *Ba*CYP106A6 are shown above the aligned sequence. Multiple sequence alignment was conducted using ClustalX [41] and edited using GeneDoc. (**B**) The active site of CYPs. Electrostatic surfaces and charge distribution of the proteins were analyzed using the Adaptive Poisson–Boltzmann Solver [42]. Arg295 and the corresponding residues from CYPs are represented as different-colored sticks.

A DALI structural homology search revealed that the *Ba*CYP106A6 structure showed high structural similarity (Z-score:54.1) to *Bm*CYP106A2 from *B. megaterium* ATCC13368 (PDB code: 4YT3 and 5IKI), *Bm*CYP109A2 from *B. megaterium* (PDB code 5OFQ), and *Bm*CYP109E1 from *B. megaterium* (PDB code 5L92) (Table 2). Multiple sequence alignment indicated that the Arg295 residue is unique to the *Ba*CYP106A6 sequence, and other CYP106 and CYP109 homologs do not have this positively charged residue at the corresponding position (Fig. 6A). The substrate-binding site of *Ba*CYP106A6 is mainly composed of hydrophobic residues and interacts with the backbone of the steroids. However, the existence of Arg295 generates a unique environment in the substrate-binding site (*e.g.*, changing the charge distribution and size of the substrate access channel) (Fig. 6B). Thus, it is thought that *Ba*CYP106A6 may have a different substrate-binding mode and specificity controlled by Arg295 compared with those of other CYP106 and CYP109 proteins.

**Fig. 7:**
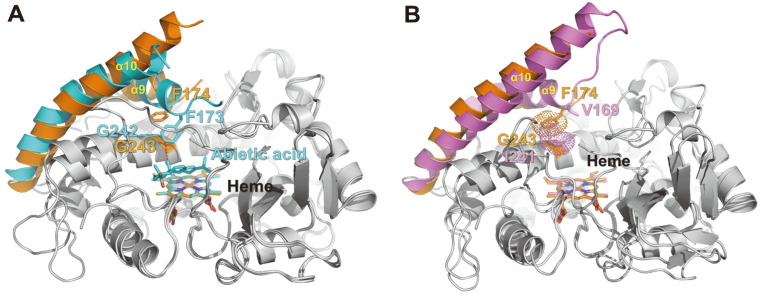
Structural comparison of *Ba*CYP106A6 with other CYPs. (**A**) Superposition of *Ba*CYP106A6 and abietic acid-bound *Ba*CYP106A2 (PDB code 5IKI). The α9, α10 helices, loop regions of *Ba*CYP106A6, and the corresponding regions of *Ba*CYP106A2 are shown as ribbon diagrams colored orange and cyan, respectively. The phenylalanine residues of *Ba*CYP106A6 and *Ba*CYP106A2 are represented by stick models. Bound heme molecules and abietic acid are also represented by stick models. (**B**) Superposition of *Ba*CYP106A6 and *Bm*CYP109E1 (PDB code 5L90). *Bm*CYP109E1 is colored violet. The residues Val169 and Ile241 of *Bm*CYP109E1 are represented by violet stick models. Phe174 of *Ba*CYP106A6 and Ile241 of *Bm*CYP109E1 occupy the same position in each substrate-binding pocket.

Besides the Arg295 residue, CYP106 and CYP109 proteins have several striking residue composition differences in the substrate-binding pocket. In *Bm*CYP106A2 (PDB code 5IKI), Phe174, located in the α9–α10 loop region, forms hydrophobic interactions with the bound substrate (abietic acid). These interactions may induce α9–α10 loop and α9 helix movements inside the substrate-binding pocket. This Phe residue is highly conserved in the CYP106 protein sequences (Fig. 7A). On the other hand, in *Bm*CYP109E1 (PDB code 5L90), this Phe is substituted for valine (V169). Previous site-directed mutagenesis experiments have shown that the V169A mutant of *Bm*CYP109E1 almost lost its ability to produce 16β-hydroxytestosterone. Another residue is Gly243, which is located on the α12 helix of *Ba*CYP106A6. Although CYP106 proteins have glycine at this position, CYP109 proteins have isoleucine. The I241A mutant of *Bm*CYP109E1 was almost completely deprived of 16β-hydroxytestosterone production. Interestingly, in the *Ba*CYP106A6 structure, the Phe174 side chain occupied the space of the Ile241 side chain in *Bm*CYP109E1 (Fig. 7B). Taken together, these residues located in the substrate-binding pocket are crucial for distinguishing the substrate specificities of CYP106 and CYP109 proteins. In addition, there are sequence differences between the α4–α5 loop and α9–α10 loop regions. Although direct structural comparisons are impossible due to the partial disorder in these regions, these loop regions are also thought to be important for the substrate-binding and specificity difference between CYP106 and CYP109 proteins.

In this study, we isolated the *Ba*CYP106A6 enzyme for the first time and investigated its function and structure. Our goal was to characterize its hydroxylating activity and analyze its structure to provide new information on steroid hydroxylases. Based on the *Ba*CYP106A6-associated bioconversion results, steroids can be divided into active steroids with high catalytic activity and less active steroids. The less active steroids have the hydroxyl group at 11β and the larger side chain at 17β of steroids in common. Furthermore, the crystal structure revealed that *Ba*CYP106A6 has a unique Arg295 residue, which is part of the constituent residues of the substrate-binding site, and possibly interacts with these functional groups. Therefore, this residue is considered an important target for substrate reactivity. Although additional structural investigations of *Ba*CYP106A6 complexed with active steroids and mutational studies are needed, this finding provides insight into the specificity and regioselectivity of *Ba*CYP106A6 hydroxylation. In addition, we provide fundamental information for future mutational studies on altering the hydroxylation of steroids and changing product yield.

Biochemical and structural investigations revealed a substrate preference for *Ba*CYP106A6. Furthermore, structural analyses and molecular alignment–energy minimization showed how *Ba*CYP106A6 distinguishes specific steroids.

## Supplemental Materials

Supplementary data for this paper are available on-line only at http://jmb.or.kr.

## Figures and Tables

**Table 1:** X-ray diffraction data collection and refinement statistics.

Data set	*Ba*CYP106A6

X-ray source	BL-5C beamline
Space group	*P*2_1_
Unit-cell parameters (Å, °)	a=53.335, b=98.997, c=92.99, α=γ=90, β=106.412
Wavelength (Å)	0.9794
Resolution (Å)	50.00–2.80 (2.85–2.80)
Total reflections	142028
Unique reflections	22,227 (1,192)
Average I/σ (I)	31.0 (5.0)
*R*_merge_^a^	0.126 (0.471)
Redundancy	6.4 (7.4)
Completeness (%)	99.1 (97.0)

Refinement	

Resolution range (Å)	37.59-2.80 (2.94-2.80)
No. of working set reflections	22,146 (3,161)
No. of test set reflections	1,011 (122)
No. of atoms	6,309
No. of water molecules	58
*R*_cryst_^b^	0.22 (0.28)
*R*_free_^c^	0.27 (0.39)
r.m.s. bond length (Å)	0.013
r.m.s. bond angle (°)	1.667
Average B value (Å^2^) (protein)	42.28
Average B value (Å^2^) (solvent)	36.55

Ramachandran plot	

Favored (%)	96.08
Allowed (%)	3.13
Outliers (%)	0.78

^a^*R*_merge_ = Σ | <I> - I | /Σ<I>.

^b^*R*_cryst_ = Σ | |Fo| - |Fc| | /Σ|Fo|.

^c^*R*_free_ calculated with 5% of all reflections excluded from refinement stages using high-resolution data.

Values in parentheses refer to the highest-resolution shells.

**Table 2:** Structural homolog search results for *Ba*CYP106A6 from a DALI search (DALI-Lite server).

Protein	PDB code	DALI Z-score	UniProtKB code	Sequence % ID with *Ba*CYP106A6 (aligned residue number)	Reference
Abietic acid-bound CYP106A2 (*B. megaterium*)	5IKI	54.1	Q06069	65 (379/399)	[28]
CYP109A2 (*B. megaterium*)	5OFQ	45.2	D5DF88	36 (373/387)	[23]
Corticosterone bound CYP109E1 (*B*. *megaterium*)	5L91	45.1	D5DKI8	42 (368/391)	[43]
Cytochrome P450 TbtJ1 (*Thermobispora bispora*)	5VWS	43.4	D6Y4Z8	36 (367/376)	[44]
CYP109B1 (*Bacillus subtilis*)	4RM4	43.3	O34374	40 (351/364)	[45]
Mycinamicin bound Cytochrome P450 (*Micromonospora griseorubida*)	2Y5N	43,0	Q59523	30 (370/400)	[46]
Cytochrome P450 MoxA (*Nonomuraea recticatena*)	2Z36	42.5	Q2L6S8	27 (373/404)	[47]
Cytochrome P450 OleP from (*Streptomyces antibioticus*)	5MNV	41.9	Q59819	29 (367/397)	[48]
