# Supplementary material for: Crystal Structure and Biochemical Analysis of a Cytochrome P450 Steroid Hydroxylase (BaCYP106A6) from Bacillus Species
Source: J Microbiol Biotechnol. 2022 Dec 12;33(3):387–97. doi: 10.4014/jmb.2211.11031 (PMC10084758; doi:10.4014/jmb.2211.11031)

J. Microbiol. Biotechnol.
https://doi.org/10.4014/jmb.2211.11031

Supplementary Figures

Crystal structure and biochemical analysis of a cytochrome P450 steroid hydroxylase (*Ba*CYP106A6) from *Bacillus* species

Ki-Hwa Kim[1†], Hackwon Do[2,3†], Chang Woo Lee[2†], Pradeep Subedi[1], Mieyoung Choi[4], Yewon Nam[2] Jun Hyuck Lee[2,3], and Tae-Jin Oh[1,4,5]*

[1]Department of Life Science and Biochemical Engineering, Sunmoon University, Asan 31460, Republic of Korea

[2]Research Unit of Cryogenic Novel Materials, Korea Polar Research Institute, Incheon 21990, Republic of Korea

[3]Department of Polar Sciences, University of Science and Technology, Incheon 21990, Republic of Korea.

[4]Department of Pharmaceutical Engineering and Biotechnology, Sunmoon University, Asan 31460, Republic of Korea.

[5]Genome-based BioIT Convergence Institute, Asan 31460, Republic of Korea.

[†]These authors contributed equally to this work.

***Correspondence**

Dr. Jun Hyuck Lee,

E-mail: tjoh3782@sunmoon.ac.kr.

Figure S1. Characterization of purified *Ba*CYP106A6. (**A**) The UV-visible spectrum of *Ba*CYP106A6. The spectrum of the oxidized ferric form of CYP106A6 is shown. Insets show enzymes purified by SDS-PAGE with protein markers. (**B**) The three states of *Ba*CYP106A6. The solid line indicates the oxidized ferric (Fe^{+3}) state of the heme iron of *Ba*CYP106A6, whereas the long-dotted line indicates ferrous (Fe^{+2}) derived from the addition of sodium dithionite. The short-dotted line represents the result of adding carbon monoxide to the heme iron.

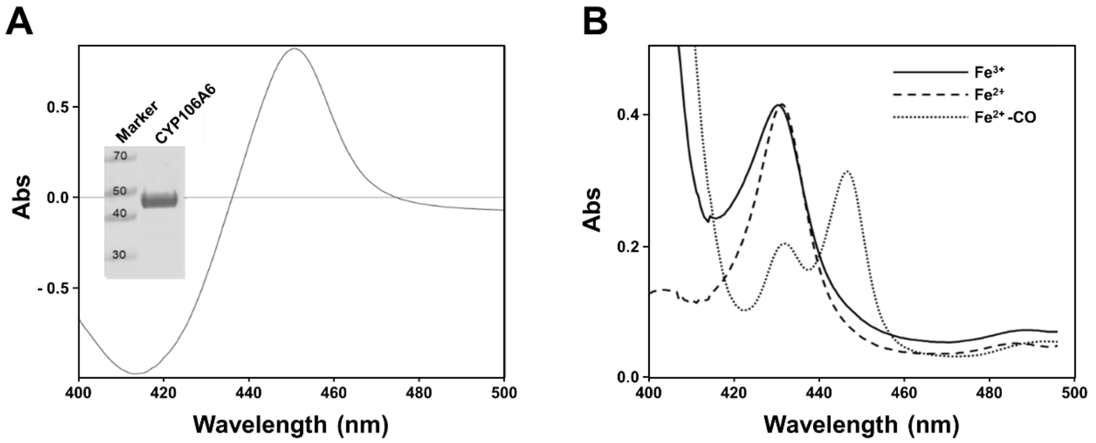

Figure S2. Spectral determination of interaction between steroids and *Ba*CYP106A6. The steroids were diluted in the range of 0–500 μm and used for the assay. The UV-visible absorbance spectra were measured between 350 and 500 nm in the presence of different concentrations of steroids and plotted in different colors. The concentration of *Ba*CYP106A6 was 1 μM in 50 mM potassium buffer (pH 7.4).

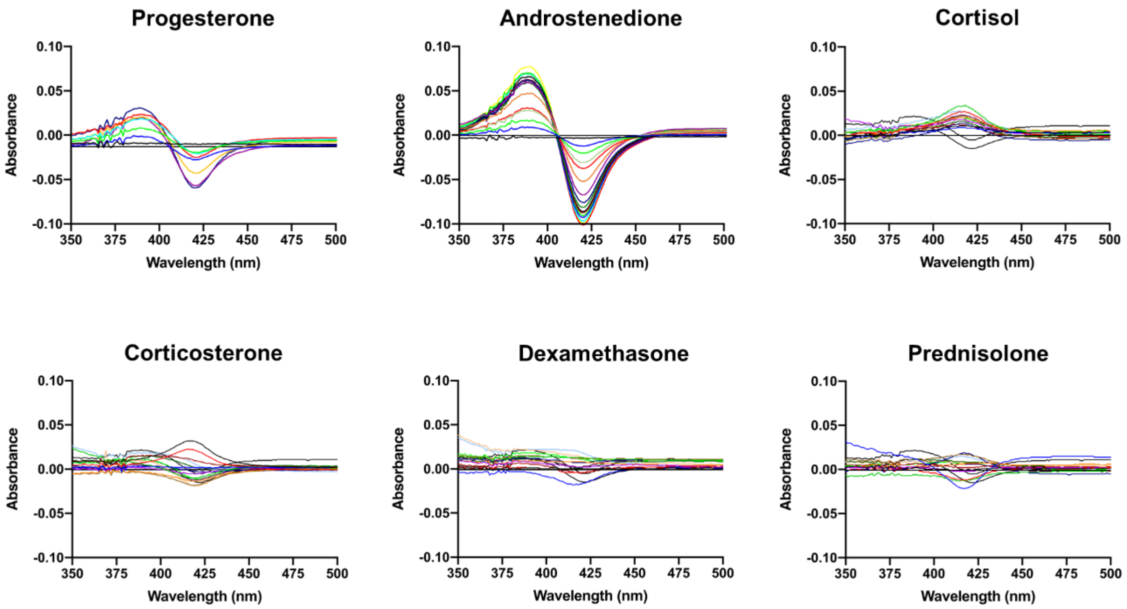

Figure S3 Oligomeric state of *Ba*CYP106A6. (**A**) Size-exclusion chromatography of *Ba*CYP106A6 was performed at 280 nm. A Superdex 200 10/300 GL column connected to an ÄKTA Avant system (Cytiva, Marlborough, MA, USA) was used. (**B**) The protein standard mix ranged from 15 to 600 kDa (cat. No. 69385-30MG; Sigma-Aldrich) was used to generate a standard curve under the same conditions. The calculated molecular mass of *Ba*CYP106A6 was 46.8 kDa.

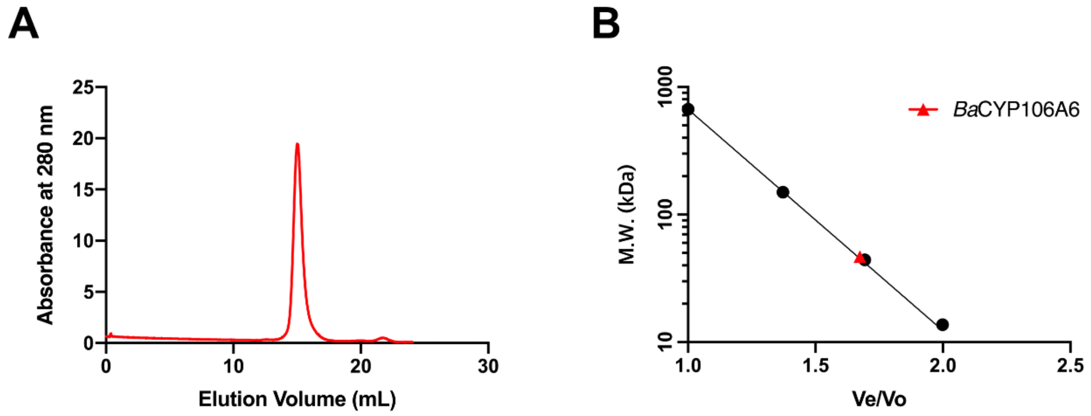

Figure S4 Stereo view of the *Ba*CYP106A6 structure centered in the substrate-binding pocket. The 2*Fo*–*Fc* electron-densit map (contoured at 1σ) around Arg295 is shown.

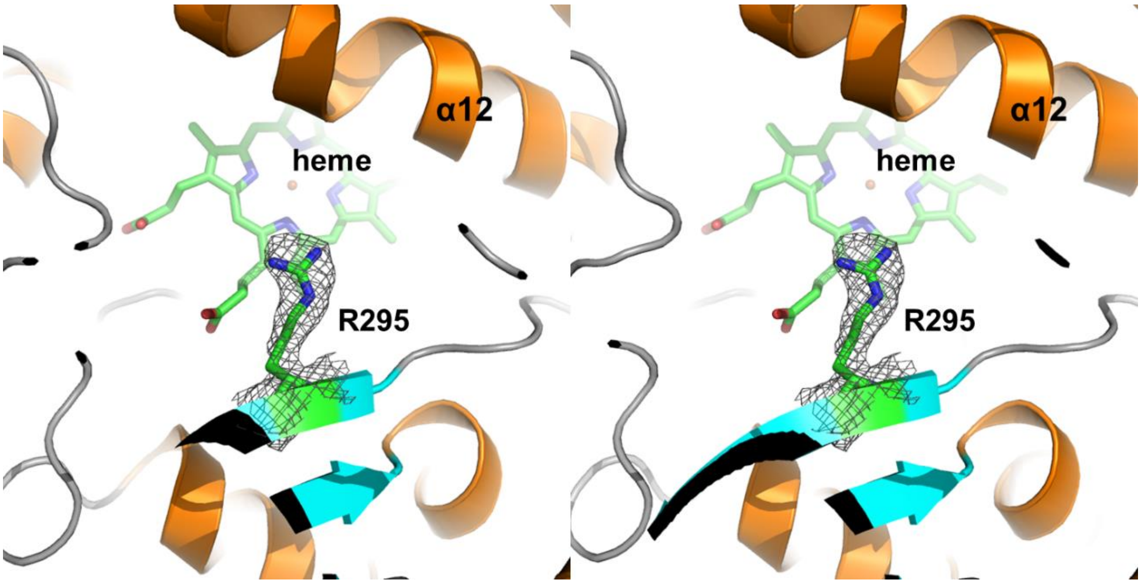

Supplement: Supplementary file 1 [file jmb-33-3-387-supple.pdf]
